# The effect of stent graft curvature on the hemodynamic displacement force after abdominal aortic aneurysm endovascular repair

**DOI:** 10.1098/rsos.230563

**Published:** 2023-07-05

**Authors:** Moshe Brand, Bar Yoel, Eran Eichler, Chen Speter, Moshe Halak, Gil Marom

**Affiliations:** ^1^ Ariel University, Ariel, Israel; ^2^ Department of Vascular Surgery, The Chaim Sheba Medical Centre, Tel Hashomer, Israel; ^3^ Tel Aviv University, Ramat Aviv, Tel Aviv, Israel

**Keywords:** abdominal aortic aneurysm (AAA), stent graft (SG), endovascular aortic aneurysm repair (EVAR), displacement force (DF), migration, centreline curvature (CLC)

## Abstract

Endovascular aortic aneurysm repair is a minimally invasive procedure with low mortality and morbidity. Clinical studies have revealed that a displacement force (DF) can cause stent graft (SG) migration in some circumstances requiring repeated intervention. This study aims to determine the relationship between the SG curvature and the calculated DF from four patient-specific computational fluid dynamics models. The SG's curvature was defined according to the centrelines of the implanted SG's branches. The centrelines were defined as either intersecting or separated lines. The centreline curvature (CLC) metrics were calculated based on the local curvature radii and the distances from the centrelines of idealized straight branches. The average CLC value and average variation were calculated to represent the entire graft's curvature. These CLC calculations were compared, and the method that gave the best correlation to the calculated DF was found. The optimal correlation is obtained from calculating the CLC average variation using separated centrelines and distance from straight lines, with an *R*^2^ = 0.89. Understanding the relationship between vascular morphology and DF can help identify at-risk patients before the procedure. In these cases, we can provide appropriate treatment and follow up with the patient to prevent future failure.

## 1. Introduction

While the traditional method of abdominal aortic aneurysm (AAA) repair is open surgery, in recent years, aneurysms are increasingly repaired endovascularly by inserting a stent graft (SG) into the aorta in a minimally invasive procedure [1]. Despite the low mortality and morbidity of endovascular aortic aneurysm repair (EVAR), in up to 22% of all cases, there is SG migration (SGM) [2]. Clinical studies have shown that the risk of SGM and the need for repeated interventions increase in cases with a relatively large diameter of the aneurysm neck or a curved structure [3–5]. Experimental studies and analytical mechanics models showed that these anatomical parameters affect the displacement or drag force (DF), which fosters SGM [6–8]. Therefore, it can be assumed that if the DF acting on the stent walls is greater than the fixation to the arterial wall, the stent may migrate out of its place and cause an endoleak, widening of the aneurysm or even rupture.

Computational mechanics models can assist in accurately assessing the artery's condition after SG insertion [9–11]. The effect of different geometric parameters on EVAR results was previously investigated using numerical methods, including parameters such as chimney length [12,13], SG oversizing, aortic neck calcification [14], aneurysm neck angle [15] and SG bifurcation geometry [16,17]. Displacement force was calculated in computational fluid dynamics (CFD) studies, with both representative anatomies [18,19] and patient-specific geometries [20–22].

Several computational studies attempted to correlate the anatomical parameters of SG to the hemodynamic DFs. Molony *et al*. [23] reconstructed four SGs of patients and demonstrated that the ratio of the cross-section areas at the inlet and outlets of the SG is well correlated with the DF. Figueroa *et al*. [24] investigated the effect of SG curvature on the magnitude and direction of the DFs and showed that increased curvature elevated the magnitude of the lateral DF. In their following work [25], they found that the DF orientation is perpendicular to the greatest SG curvature, but no correlation to the size of the force was found. Georgakarakos *et al*. [26] compared the hemodynamic behaviour of a model with a ‘crossed limbs’ configuration. They showed that SG curvature had little effect on the DF because of the more substantial changes in the bifurcation and iliac forces. Kandail *et al*. [27] examined six patients with various AAA morphologies. The geometries in the pre-and post-operative stages were reconstructed for each patient from computed tomography (CT) images, and appropriate boundary conditions were applied. While they found a strong correlation between DF magnitude and anterior/posterior aortic neck angle (*R*^2^ = 0.97), they only found a weak correlation with the lateral neck angle (*R*^2^ = 0.32). Tasso *et al*. [28] explored two different stent grafts in 20 patients, focusing on the ratio between DFs and cross-sectional area. The curvature was calculated from centreline splines, but no correlation was found between the curvature and the DF. Still, a following study [29] suggested that DF is influenced by the in-stent helical blood flow patterns, which is probably related to the curvature. Finally, Belvroy *et al*. [29] showed that higher DFs in the descending thoracic aorta are associated with a greater tortuosity angle and that the vector's direction of the DF is changed from the proximal to the distal part. However, to our knowledge, no previous study has correlated the curvature of a specific patient's SG with the DFs.

In this study, we aim to determine the relationship between the curvature of the SG and the calculated DF from patient-specific CFD models. We used geometric models of four patients, which were reconstructed according to CT scans taken after the SG insertion.

A better understanding of the relationship between vascular morphology and DF can help identify patients at risk before the procedure and provide appropriate treatment, and post-procedure follow-up to prevent future failure.

## 2. Methods

In this research, four three-dimensional CFD models were created to study the hemodynamics inside abdominal SGs. Models' results were used to evaluate the DF exerted on the grafts as a proxy for the possible risk of SGM.

**Figure 1.**
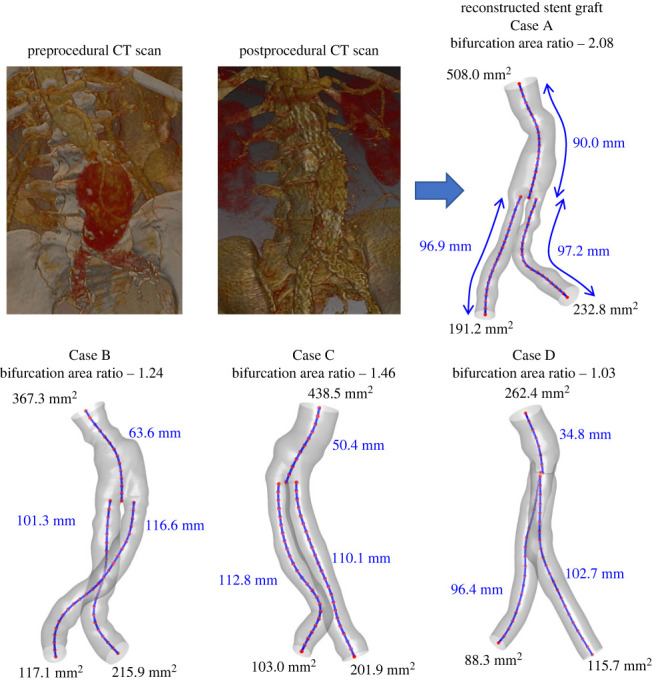
Description of four patient-specific SGs with dimensions of the branches' inlet and outlet areas and centreline lengths. For each case, the bifurcation area ratios are also given, defined as the ratio between the inlet area and the total outlet area near the bifurcation. Pre- and post-procedural CT scans are also presented for Case A.

To create the fluid domain, patient-specific geometries of the SGs were reconstructed in the region of the descending aorta between the renal arteries and the iliac artery bifurcation. Routine post-procedural three-dimensional CT scans, with a resolution of 0.5 × 0.5 × 0.625 mm^3^, were obtained from patients that underwent SG implantation in Sheba Medical Centre (this study was approved by the institutional ethics committee). The geometries were reconstructed with SimVascular open-source software [30] by finding the centreline of three branches of the SG, segmenting sections that are normal to these lines, and extracting the volumetric geometry by segmental lofting. Since this is a CFD study, the geometry of the lumen was reconstructed, and it is independent of the SG type. Figure 1 shows the four geometries with representative dimensions, including branch lengths. Finally, these geometries were smoothed in Autodesk Meshmixer (Mill Valley, CA, USA). These geometries were meshed with tetrahedral cells in ANSYS Meshing and converted to polyhedral cells in ANSYS Fluent (Canonsburg, PA, USA), resulting in meshes with at least 600 000 cells based on a mesh sensitivity study.

The blood was assumed to be Newtonian $\left(\rho =1050\;\mathrm{kg}\;m^{-3},\;\mu =0.0035\;N\;s\;m^{-2}\right)$ and the flow was assumed to be incompressible and laminar, a reasonable assumption in large arteries such as the abdominal aorta and for drag calculations [8,31,32]. Steady-state simulations with the same peak systolic boundary conditions were defined to model the worst-case scenario in all models. Similar studies also used the same boundary conditions for all patients [21,26,27,33]. A peak systolic mass flow of 0.2 l s^−1^ in the abdominal aorta and a peak pressure of 16 kPa in the iliac arteries were defined based on previously published measurements from a patient with AAA [32]. No-slip conditions were imposed on the walls of the domain. The finite volume model was used to discretize and solve the mass and momentum conservation equations using ANSYS Fluent. The iterative convergence criteria were based on a threshold of residuals lower than 1 × 10^−6^.

**Figure 2.**
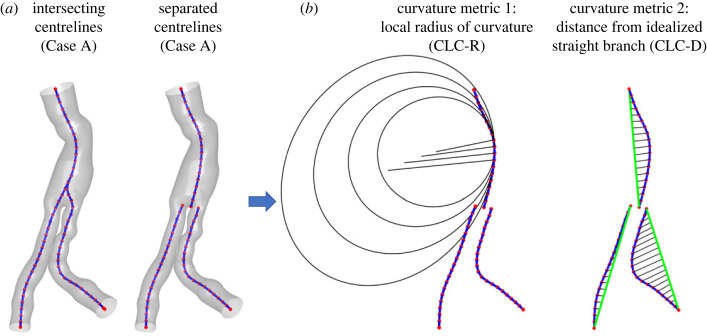
Schematic description of Case A with visualization of two centreline definitions, intersected and separated (*a*), and the two suggested curvature metrics, which are demonstrated on the separated centrelines (*b*).

The total DF that the blood flow applies on the SG is a common metric of SGM risk, and therefore it was previously calculated for endovascular treatments for AAA [34]. The total DF is a global metric that depicts the flow traction by integrating the flow stress tensor, both pressure and wall shear stress (WSS) components, to which the graft's wall is exposed. The resultant DF can provide information on the magnitude and direction, but it cannot be used for studying more local phenomena, like the local distribution and its relation to the patient-specific implantation configuration. Since this kind of phenomenon is the focus of the current study, we suggest quantifying the centreline curvature (CLC) of the implanted graft and determining if it is related to the DF. Two methods are suggested to define and quantify the curvature of the graft. Both are based on the centreline of the graft, and both were calculated for intersecting separated centrelines (figure 2). The difference between the centreline definitions is the location where the aorta splits into the iliac segments. In the ‘intersecting centrelines’, the two iliac segments ascend to the aorta, and the three lines connect at a common point. On the other hand, in the ‘separated centrelines', the aortic line reaches the cross-section, and the two iliac segments are represented by two lines beginning at the same cross-section.

The first suggested CLC metric is based on the local radius of curvature, abbreviated as CLC-R, as demonstrated in figure 2*b*. Centrelines were smoothed with a cubic spline, and discrete points along them were redistributed with 5 mm spacing to avoid curvature inconsistencies. Since the centrelines are defined by their points, it is possible to look at each subset of three consecutive points and find the radius of the circular arc that connects them and the reciprocal curvature of this radius. Therefore, a perfectly straight line will have an infinite radius and a curvature approaching zero. In our case, we are interested not only in these values but mainly in their distribution along the centreline. However, when representing the entire graft, the average curvature value can be misleading since it may show very low values even for meandering lines. Therefore, in this study, we preferred to look at the variations of the values. Still, a major limitation of this metric is that, even for similar curvature radii, the curvature's centre might substantially move, and even a metric of low curvature alteration may represent a highly meandering line.

The second suggested metric is based on comparing each branch to a similarly located idealized straight branch, as demonstrated in figure 2*b*. For this purpose, three main branches were defined: the abdominal aorta above the bifurcation, the right and the left common iliac arteries. For each branch, a comparable, ideally straight branch was defined as a straight line connecting its entrance and exit centres. Since the distance of each centreline point from this idealized line is mathematically well defined, it can indicate how far the anatomical branch is from the ideal one. Therefore, the second metric represents distance and is abbreviated as CLC-D. Similar to the first metric, the average value of this distance along the curves and the average variation are calculated to represent the entire graft.

In addition to these two suggested metrics, geometric qualities considered in this study as indicators of DF are the previously suggested area ratio between the inlet and outlets of the bifurcation region [8,23] and the length and surface area of the graft. The latter two directly contribute to the DF magnitude because of its integral nature. Correlations between the DF and all the metrics were found, and their quality was evaluated by the coefficients of determination (*R*^2^).

## 3. Results

### 3.1. Local drag force distribution

**Figure 3.**
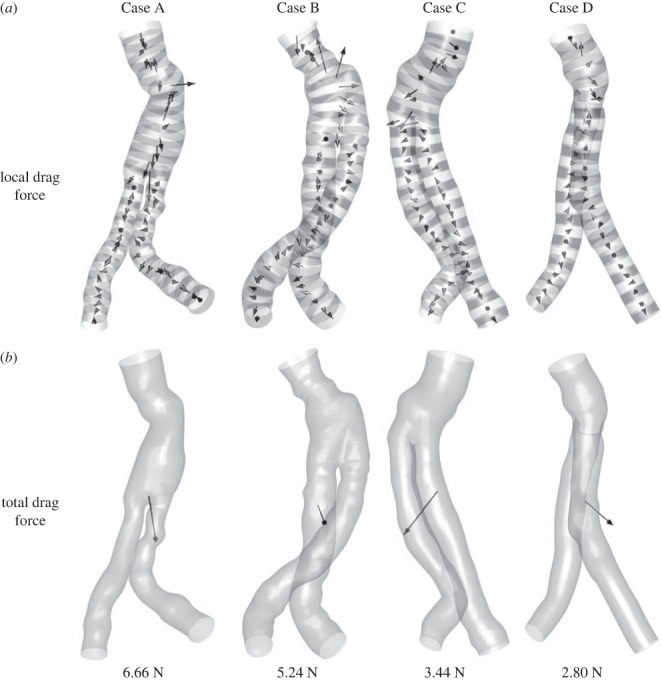
Comparison of local DF on sections along the centrelines (*a*) and total DF (*b*) in the four cases.

Local DFs were calculated on consecutive slices of 5 mm thickness (figure 3*a*). The arrows in each slice depict the force vectors that contribute to calculating the total DF value, shown in figure 3*b* in descending order, with a visual representation of the direction and size of the vectors. It can be seen that the largest local DFs are in the regions of high curvature and at the bifurcation. Naturally, higher local drag forces must also be found in regions with a larger surface area or slice perimeter. Therefore, the lowest total DF in Case D can be explained by its significantly smaller sheath area (see dimensions in figure 1).

### 3.2. Stent graft curvature calculation and the effect of stent graft curvature on displacement force

**Figure 4.**
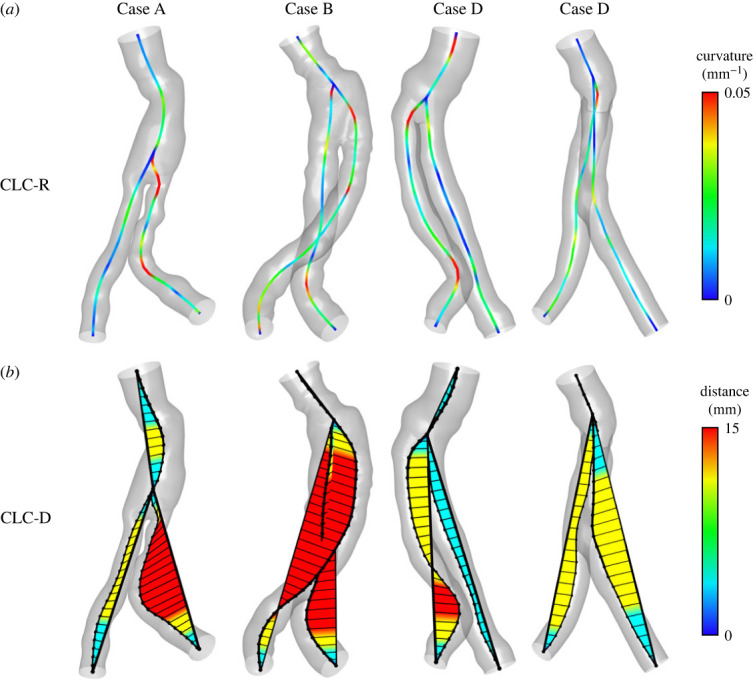
Centreline curvature (CLC) distributions along intersecting centrelines according to the two methods: CLC-R (*a*) and CLC-D (*b*).

**Figure 5.**
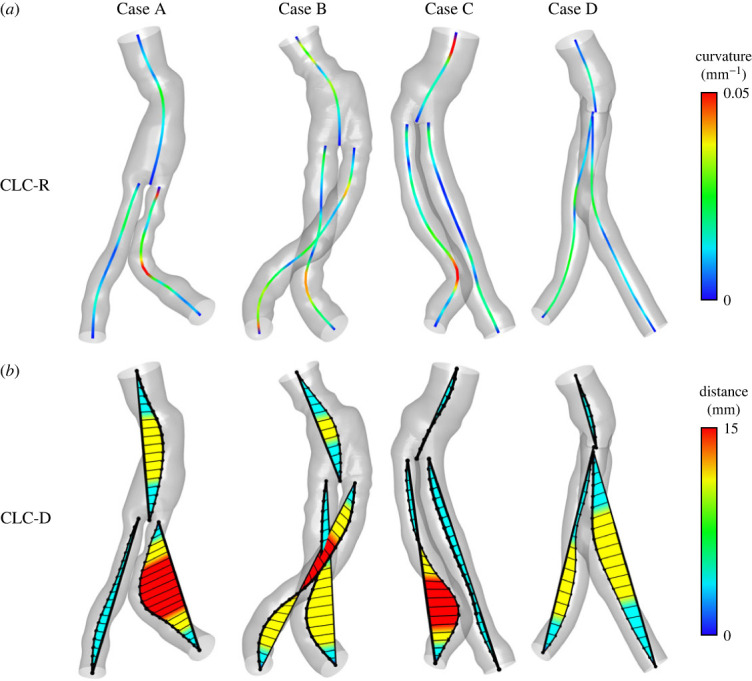
Centreline curvature (CLC) distributions along separated centrelines according to the two methods: CLC-R (*a*) and CLC-D (*b*).

Different approaches to defining the curvature value were calculated to find an optimal correlation between the DFs and the curvature of the SG structure. The results are presented on centrelines that intersect (figure 4) or separate (figure 5) in the bifurcation area, as described in figure 2.

The curvature of the centrelines was calculated using a local radius of curvature (CLC-R) and distance from a straight branch (CLC-D), as described in figures 4 and 5 in the upper and lower rows, respectively. The colour of the centreline represents the curvature values calculated by the CLC-R method. The area's colour between the centreline and a straight line and the distance represent the curvature values calculated by the CLC-D method.

It can be seen that the CLC-R curvature value near the bifurcation of the aortic segment to the iliac segments is higher with the intersecting lines method (figure 4*a*) than with the separate lines method (figure 5*a*). This result makes sense because the separation of the centrelines aligns them in the bifurcation area and reduces the CLC-R values.

**Figure 6.**
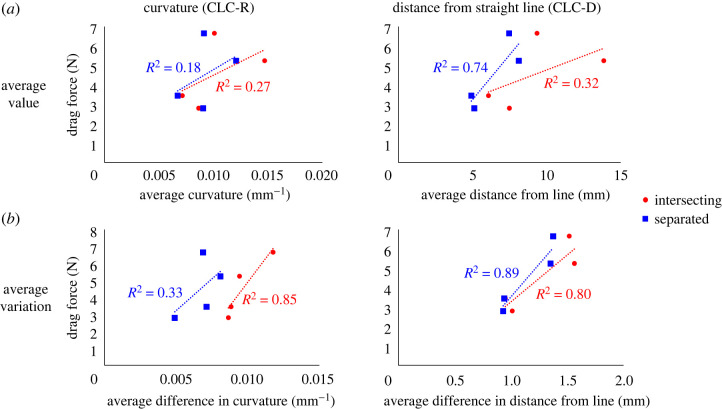
Total DF magnitudes as a function of the average CLC metrics (*a*) and the average variation of the CLC metrics (*b*). The two considered CLC metrics, CLC-R and CLC-D, are presented in the left and right columns, respectively. Results are presented for intersecting (red) and separated (blue) centrelines.

A similar phenomenon also occurs when calculating curvature values using the CLC-D method. In this case, the separation of the centrelines reduces the distances measured by this method, as described in figures 4*b* and 5*b*. This result is reflected in figure 6, where red points represent values calculated using the intersecting lines method, and blue squares represent values calculated using the separate lines method. In most cases, the red points are located to the right of the blue squares, meaning their curvature values are greater, and in the only exception, the red and blue points are very close to each other.

The curvature with the CLC-D method can especially emphasize which segments appear to be straight. For example, in the case of D in the left iliac artery (figures 4 and 5, bottom row, right branch), high curvature and local DF magnitudes can be seen (figure 3).

Two approaches were defined to calculate the weighted curvature to link the magnitudes of the total DF and the overall SG curvature. One was calculated as the average of the curvature values in all segments, and the other as the average of the changes in curvature between adjacent segments.

The relationship between the DF magnitudes and the curvature in the different methods is shown by *R*^2^, a coefficient of determination (figure 6). The curvature quantifications using the CLC-R and CLC-D methods are shown in the left and right columns, respectively. In figure 6*a*, the curvature was calculated as the average of the curvature values, and in figure 6*b* as the average of the changes. *R*^2^ was calculated to represent how much the results are linearly correlated.

The best correlation between the total DF values and the endograft's weighted curvature is obtained by combining the separate centrelines with the CLC-D method and considering the average variation in the curvature, resulting in *R*^2^ = 0.89 with a *p*-value of 0.045 (figure 6*b*, right panel). In all cases, the average variation method obtained a better fit than the average value method (figures 6*b* and 6*a*, respectively). Moreover, in the calculation based on the average variation, *R*^2^ is above 0.8 in three out of the four combinations of methods. By contrast, in the average values, *R*^2^ never exceeded 0.74, and in the other three combinations, it was even below 0.32. For the CLC-R index, intersecting centrelines provide better predictors (a higher *R*^2^) than separated ones, while for the CLC-D index, the opposite trend was found.

**Figure 7.**
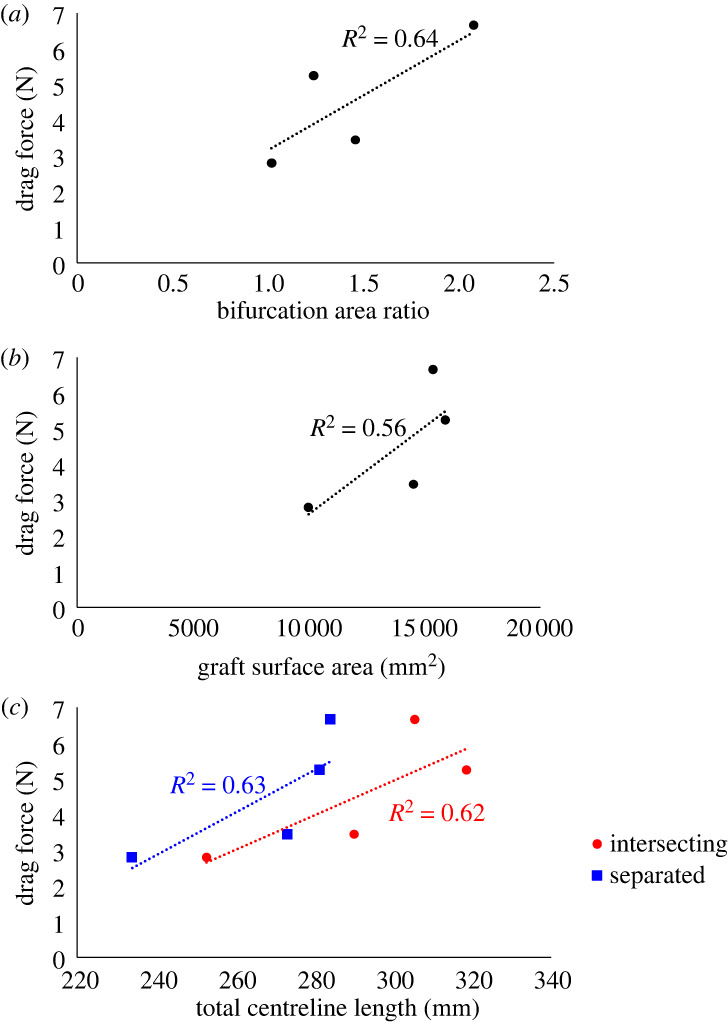
Total DF magnitudes as a function of the cross-sectional area ratio before and after the bifurcation (*a*), graft surface areas (*b*), and the total length of the centrelines (*c*). The total length results are presented for intersecting (red) and separated (blue) centrelines.

For comparison, the correlations between the DF magnitudes and additional geometric parameters were also examined. These geometric parameters were the cross-sectional areas before and after the bifurcation (figure 7*a*), the graft surface area (figure 7*b*), and the total length of the branches along the centrelines, both intersecting and separated (figure 7*c*). For all these additional parameters that were tested, significantly lower *R*^2^ values were obtained than the high values in the newly suggested parameters in this study, as shown in figure 6.

## 4. Discussion

This study examined the correlation between SG curvature and DF in four patients with distinctive endograft structures. Those distinctive structures are large bifurcation area ratio (greater than 2; Patient A), ballerina-type structure (Patient B), standard anatomical dimensions (Patient C), and extremely small ones (Patient D). In addition, Patient A is also distinguished by the long aortic portion and large cross-sectional areas at the inlet and outlets. Assuming that the cardiac output in adults is similar, the same flow rate was defined for the four patients [35–37]. This type of boundary condition also ensures that changes in the DF are a result of the flow structure and not a result of differences in the flow rate, as might happen when the common boundary conditions are defined based on pressures.

Eight combinations of several methods have been defined to assess the curvature of the endograft (figure 6). These were combinations of defining the centreline as separated or intersecting, calculating the local curvature based on the radius of curvature (CLC-R), or according to the distance from a straight line between the ends of the segment (CLC-D). The representative curvature of the entire SG was calculated as the average of the local curvatures or the average of the changes between each consecutive segment.

The results from the intersecting centrelines are always to the right of the results from the separated centrelines (red and blue in figure 6, respectively), highlighting the curvier nature of the intersecting lines (figures 4 and 5). The averaged CLC-D variance for separated centrelines yielded the best estimate of DF, with an *R*^2^ of 0.89 (*p* = 0.045) (figure 6*b*, right panel).

The average variation indices (figure 6*b*) show a notably better fit than the average value (figure 6*a*). The *R*^2^ values of the average variation are 1.2 to 3.1 times higher than those found in the fitting of the average values. This result shows that the total DF magnitude is more sensitive to changes in curvature than the average curvature, maybe because changes in curvature can be related to changes in direction.

The calculation of the curvature using the separate lines method resulted in the best results for *R*^2^ using the CLC-D method, both in the calculation of the average value (*R*^2^ = 0.74) and in the calculation of the average variation (*R*^2^ = 0.89) (figure 6, right column, blue squares). The ratio between these two values is also the smallest compared to the other methods and expresses the insensitivity of this method to the type of averaging (0.89/0.74 = 1.2). These results represent the effect of the curvature calculated by the CLC-D method on the DF values when an optimal match is obtained with separated centrelines or when the bifurcation of the centrelines is not considered.

Also, with the intersecting lines method, the best results were found for the average of the curvature changes, with *R*^2^ = 0.85 (*p* = 0.029) and *R*^2^ = 0.80 (*p* = 0.045) for CLC-R and CLC-D, respectively (figure 6*b*, red circles). A possible explanation is that with separated lines, the curvature is more accurately represented by CLC-D since the curvature in the bifurcation is meaningless. On the other hand, when the lines intersect, there is an emphasis on the bifurcation area, which is reflected in the curvature changes in both methods (CLC-R and CLC-D).

Several studies have examined the relationship between the DF and geometric parameters. Molony *et al*. examined the ratio between DF and the cross-sectional areas at the inlet and outlets in the bifurcation area. They obtained *R*^2^ = 0.72 [23], similar to the value obtained in our study using the same method (*R*^2^ = 0.64) (figure 7*a*). Other geometric parameters examined with the DF are the SG's surface area and the centrelines' total length (figure 7*b*,*c*, respectively). The *R*^2^ values obtained are 0.56, 0.62, and 0.63 and are lower than the new indices defined in this study. Therefore, calculating the curvature is a better predictor of DF than a simple geometric parameter that represents just the dimensions of the SG, probably because the curvature predominantly affects the local DF.

Although it would be interesting to compare the correlations of this study to previous studies, it is impossible to do so directly since none of the studies expressed the SG's weighted curvature relative to the calculated DF. Instead, these studies focused on geometric parameters such as angles in different two-dimensional sections, the length of SG segments, the diameter ratio of the stent graft, etc. [19–22]. Other studies dealt with the qualitative relationship of geometric parameters with the DF [12–17], proposing hypotheses linking geometric parameters and DF but not quantitatively. It can still be seen that the DF values obtained in this study (3–7 N) are in good agreement with other studies [19,21,24,25,33] and more.

In this study, we calculate the DF values for a steady state with peak systole pressure. Results obtained in previous studies showed that maximum DFs are obtained during peak systole [21,23,24,27,33]. Considering this, it can be assumed that this situation does not constitute a limitation and that it characterizes the extreme case in which maximum values of the DF are obtained. Since the DF was calculated steadily, there is no significance to the movement of the endograft wall and the stent graft, and there is no need for FSI analysis. Since a more realistic and accurate transient model with FSI will provide lower predictions of the DF, our current model can be seen as a conservative model of the DF. This study was limited to a few patients like in previous studies [10,20,25–27,32,38–40]. Expansion to additional patients with different geometries in a follow-up study will allow us to present broader conclusions with clinical significance.

To sum up, it can be concluded that the endograft's curvature is the dominant parameter in predicting drag forces acting on SG. Calculating the curvature of the endograft for a specific patient preoperatively, using the above methods, will allow identifying patients with a large DF that may cause migration. For those patients, selecting a different SG with a stronger fixation in the neck area or preferring a traditional surgery will be possible.

## Data Availability

The new data in this submission are available in the supplementary material [41].

## References

[1] Jones SM, Poole RJ, How TV, Williams RL, McWilliams RG, Brennan JA, Vallabhaneni SR, Fisher RK. 2014 Computational fluid dynamic analysis of the effect of morphologic features on distraction forces in fenestrated stent grafts. J. Vasc. Surg. **60**, 1648-56 e1. (10.1016/j.jvs.2014.08.077) 25454107

[2] England A, Garcia-Finana M, Fisher RK, Naik JB, Vallabhaneni SR, Brennan JA, McWilliams RG. 2013 Migration of fenestrated aortic stent grafts. J. Vasc. Surg. **57**, 1543-1552. (10.1016/j.jvs.2012.12.035) 23541544

[3] Igari K, Kudo T, Toyofuku T, Jibiki M, Inoue Y. 2014 Outcomes following endovascular abdominal aortic aneurysm repair both within and outside of the instructions for use. Ann. Thorac. Cardiovasc. Surg. **20**, 61-66. (10.5761/atcs.oa.12.02059) 23411843

[4] Chisci E, Kristmundsson T, de Donato G, Resch T, Setacci F, Sonesson B, Setacci C, Malina M. 2009 The AAA with a challenging neck: outcome of open versus endovascular repair with standard and fenestrated stent-grafts. J. Endovasc. Ther. **16**, 137-146. (10.1583/08-2531.1) 19456190

[5] Stather PW, Sayers RD, Cheah A, Wild JB, Bown MJ, Choke E. 2012 Outcomes of endovascular aneurysm repair in patients with hostile neck anatomy. Eur. J. Vasc. Endovasc Surg. **44**, 556-561. (10.1016/j.ejvs.2012.10.003) 23122183

[6] Roos H, Ghaffari M, Falkenberg M, Chernoray V, Jeppsson A, Nilsson H. 2014 Displacement forces in iliac landing zones and stent graft interconnections in endovascular aortic repair: an experimental study. Eur. J. Vasc. Endovasc Surg. **47**, 262-267. (10.1016/j.ejvs.2013.11.015) 24445085

[7] Mohan IV, Harris PL, Van Marrewijk CJ, Laheij RJ, How TV. 2002 Factors and forces influencing stent-graft migration after endovascular aortic aneurysm repair. J. Endovasc. Ther. **9**, 748-755. 12546574 10.1177/152660280200900606

[8] Morris L, Delassus P, Walsh M, McGloughlin T. 2004 A mathematical model to predict the in vivo pulsatile drag forces acting on bifurcated stent grafts used in endovascular treatment of abdominal aortic aneurysms (AAA). J. Biomech. **37**, 1087-1095. (10.1016/j.jbiomech.2003.11.014) 15165879

[9] Hemmler A, Lutz B, Reeps C, Kalender G, Gee MW. 2018 A methodology for in silico endovascular repair of abdominal aortic aneurysms. Biomech. Model. Mechanobiol. **17**, 1139-1164. (10.1007/s10237-018-1020-0) 29752606

[10] Perrin D, Badel P, Orgeas L, Geindreau C, Dumenil A, Albertini JN, Avril S. 2015 Patient-specific numerical simulation of stent-graft deployment: validation on three clinical cases. J. Biomech. **48**, 1868-1875. (10.1016/j.jbiomech.2015.04.031) 25979382

[11] Auricchio F, Conti M, Marconi S, Reali A, Tolenaar JL, Trimarchi S. 2013 Patient-specific aortic endografting simulation: from diagnosis to prediction. Comput. Biol. Med. **43**, 386-394. (10.1016/j.compbiomed.2013.01.006) 23395199

[12] Ben Gur H, Halak M, Brand M. 2018 Blood flow in the abdominal aorta post ‘chimney’ endovascular aneurysm repair. In Proc. 9th EUROSIM Congress on Modelling and Simulation, EUROSIM 2016, 57th SIMS Conf. on Simulation and Modelling, pp. 667-672. Linköping, Sweden: Linköping University Electronic Press.

[13] Ben Gur H, Kosa G, Brand M. 2015 Numerical analysis of the hemodynamics of an abdominal aortic aneurysm repaired using the endovascular chimney technique. In 37th Annual Int. Conf. of IEEE Engineering in Medicine and Biology Society, Milan, Italy, 25–29 August 2015, pp. 977–980. (10.1109/EMBC.2015.7318527) 26736427

[14] Hemmler A, Lutz B, Reeps C, Gee MW. 2019 In silico study of vessel and stent-graft parameters on the potential success of endovascular aneurysm repair. Int. J. Numer. Methods Biomed. Eng. **35**, e3237. (10.1002/cnm.3237) 31315160

[15] De Bock S, Iannaccone F, De Beule M, Vermassen F, Segers P, Verhegghe B. 2014 What if you stretch the IFU? A mechanical insight into stent graft instructions for use in angulated proximal aneurysm necks. Med. Eng. Phys. **36**, 1567-1576. (10.1016/j.medengphy.2014.08.003) 25217007

[16] Polanczyk A, Podyma M, Trebinski L, Chrzastek J, Zbicinski I, Stefanczyk L. 2016 A novel attempt to standardize results of CFD simulations basing on spatial configuration of aortic stent-grafts. PLoS ONE **11**, e0153332. (10.1371/journal.pone.0153332) 27073907 PMC4830540

[17] Polanczyk A, Piechota-Polanczyk A, Stefanczyk L. 2017 A new approach for the pre-clinical optimization of a spatial configuration of bifurcated endovascular prosthesis placed in abdominal aortic aneurysms. PLoS ONE **12**, e0182717. (10.1371/journal.pone.0182717) 28793343 PMC5549977

[18] Avrahami I, Brand M, Meirson T, Ovadia-Blechman Z, Halak M. 2012 Hemodynamic and mechanical aspects of fenestrated endografts for treatment of abdominal aortic aneurysm. Eur. J. Mech. B Fluids. **35**, 85-91. (10.1016/j.euromechflu.2012.03.010)

[19] Li Z, Kleinstreuer C. 2006 Analysis of biomechanical factors affecting stent-graft migration in an abdominal aortic aneurysm model. J. Biomech. **39**, 2264-2273. (10.1016/j.jbiomech.2005.07.010) 16153654

[20] Leung JH, Wright AR, Cheshire N, Crane J, Thom SA, Hughes AD, Xu Y. 2006 Fluid structure interaction of patient specific abdominal aortic aneurysms: a comparison with solid stress models. Biomed. Eng. Online **5**, 33. (10.1186/1475-925X-5-33) 16712729 PMC1488849

[21] Stefanov F, McGloughlin T, Morris L. 2016 A computational assessment of the hemodynamic effects of crossed and non-crossed bifurcated stent-graft devices for the treatment of abdominal aortic aneurysms. Med. Eng. Phys. **38**, 1458-1473. (10.1016/j.medengphy.2016.09.011) 27773830

[22] Molony DS, Callanan A, Kavanagh EG, Walsh MT, McGloughlin TM. 2009 Fluid-structure interaction of a patient-specific abdominal aortic aneurysm treated with an endovascular stent-graft. Biomed. Eng. Online **8**, 24. (10.1186/1475-925X-8-24) 19807909 PMC2764714

[23] Molony DS, Kavanagh EG, Madhavan P, Walsh MT, McGloughlin TM. 2010 A computational study of the magnitude and direction of migration forces in patient-specific abdominal aortic aneurysm stent-grafts. Eur. J. Vasc. Endovasc Surg. **40**, 332-339. (10.1016/j.ejvs.2010.06.001) 20573524

[24] Figueroa CA, Taylor CA, Yeh V, Chiou AJ, Zarins CK. 2009 Effect of curvature on displacement forces acting on aortic endografts: a 3-dimensional computational analysis. J. Endovasc. Ther. **16**, 284-294. (10.1583/08-2667.1) 19642787 PMC2793567

[25] Figueroa CA, Taylor CA, Yeh V, Chiou AJ, Gorrepati ML, Zarins CK. 2010 Preliminary 3d computational analysis of the relationship between aortic displacement force and direction of endograft movement. J. Vasc. Surg. **51**, 1488-1497. (10.1016/j.jvs.2010.01.058) 20488325 PMC2874723

[26] Georgakarakos E, Xenakis A, Manopoulos C, Georgiadis GS, Tsangaris S, Lazarides M. 2013 Geometric factors affecting the displacement forces in an aortic endograft with crossed limbs: a computational study. J. Endovasc. Ther. **20**, 191-199. (10.1583/1545-1550-20.2.191) 23581761

[27] Kandail H, Hamady M, Xu XY. 2014 Patient-specific analysis of displacement forces acting on fenestrated stent grafts for endovascular aneurysm repair. J. Biomech. **47**, 3546-3554. (10.1016/j.jbiomech.2014.08.011) 25267572

[28] Tasso P, Raptis A, Matsagkas M, Lodi Rizzini M, Gallo D, Xenos M, Morbiducci U. 2018 Abdominal aortic aneurysm endovascular repair: profiling post-implantation morphometry and hemodynamics with image-based computational fluid dynamics. J. Biomech. Eng. **140**, 111003. (10.1115/1.4040337) 30029263

[29] Belvroy VM, Romarowski RM, van Bakel TMJ, van Herwaarden JA, Bismuth J, Auricchio F, Moll FL, Trimarchi S. 2020 Impact of aortic tortuosity on displacement forces in descending thoracic aortic aneurysms. Eur. J. Vasc. Endovasc Surg. **59**, 557-564. (10.1016/j.ejvs.2019.09.503) 31924459

[30] Updegrove A, Wilson NM, Merkow J, Lan H, Marsden AL, Shadden SC. 2017 Simvascular: an open source pipeline for cardiovascular simulation. Ann. Biomed. Eng. **45**, 525-541. (10.1007/s10439-016-1762-8) 27933407 PMC6546171

[31] Liu X, Fan Y, Deng X, Zhan F. 2011 Effect of non-newtonian and pulsatile blood flow on mass transport in the human aorta. J. Biomech. **44**, 1123-1131. (10.1016/j.jbiomech.2011.01.024) 21310418

[32] Molony DS, Callanan A, Morris LG, Doyle BJ, Walsh MT, McGloughlin TM. 2008 Geometrical enhancements for abdominal aortic stent-grafts. J. Endovasc. Ther. **15**, 518-529. (10.1583/08-2388.1) 18840041

[33] Tasso P, Lodi Rizzini M, Raptis A, Matsagkas M, De Nisco G, Gallo D, Xenos M, Morbiducci U. 2019 In-stent graft helical flow intensity reduces the risk of migration after endovascular aortic repair. J. Biomech. **94**, 170-179. (10.1016/j.jbiomech.2019.07.034) 31421805

[34] Cheng SW, Lam ES, Fung GS, Ho P, Ting AC, Chow KW. 2008 A computational fluid dynamic study of stent graft remodeling after endovascular repair of thoracic aortic dissections. J. Vasc. Surg. **48**, 303-309. (10.1016/j.jvs.2008.03.050) 18644477

[35] Amanuma M, Mohiaddin RH, Hasegawa M, Heshiki A, Longmore DB. 1992 Abdominal aorta: characterisation of blood flow and measurement of its regional distribution by cine magnetic resonance phase-shift velocity mapping. Eur. Radiol. **2**, 559-564. (10.1007/BF00187552)

[36] Taylor CA, Cheng CP, Espinosa LA, Tang BT, Parker D, Herfkens RJ. 2002 In vivo quantification of blood flow and wall shear stress in the human abdominal aorta during lower limb exercise. Ann. Biomed. Eng. **30**, 402-408. (10.1114/1.1476016) 12051624

[37] Cheng CP, Herfkens RJ, Taylor CA. 2003 Comparison of abdominal aortic hemodynamics between men and women at rest and during lower limb exercise. J. Vasc. Surg. **37**, 118-123. (10.1067/mva.2002.107) 12514587

[38] Algabri YA, Altwijri O, Chatpun S. 2019 Visualization of blood flow in aaa patient-specific geometry: 3-d reconstruction and simulation procedures. Bionanoscience **9**, 966-976. (10.1007/s12668-019-00662-8)

[39] Shek TL, Tse LW, Nabovati A, Amon CH. 2012 Computational fluid dynamics evaluation of the cross-limb stent graft configuration for endovascular aneurysm repair. J. Biomech. Eng. **134**, 121002. (10.1115/1.4007950) 23363204

[40] Domanin M, Piazzoli G, Trimarchi S, Vergara C. 2020 Image-based displacements analysis and computational blood dynamics after endovascular aneurysm repair. Ann. Vasc. Surg. **69**, 400-412. (10.1016/j.avsg.2020.07.014) 32738387

[41] Brand M, Yoel B, Eichler E, Speter C, Halak M, Marom G. 2023 The effect of stent graft curvature on the hemodynamic displacement force after abdominal aortic aneurysm endovascular repair. Figshare. (10.6084/m9.figshare.c.6700190) PMC10320339 37416831

